# Design of a machine learning model for the precise manufacturing of green cementitious composites modified with waste granite powder

**DOI:** 10.1038/s41598-022-17670-6

**Published:** 2022-08-02

**Authors:** Sławomir Czarnecki, Marijana Hadzima-Nyarko, Adrian Chajec, Łukasz Sadowski

**Affiliations:** 1 Department of Materials Engineering and Construction Processes, Wroclaw University of Science and Technology, Wybrzeze Wyspiańskiego 27, 50-370 Wrocław, Poland; 2 Faculty of Civil Engineering and Architecture Osijek, Josip Juraj Strossmayer University of Osijek, Vladimira Preloga 3, 31000 Osijek, Croatia

**Keywords:** Civil engineering, Computational methods

## Abstract

In this study, a machine learning model for the precise manufacturing of green cementitious composites modified with granite powder sourced from quarry waste was designed. For this purpose, decision tree, random forest and AdaBoost ensemble models were used and compared. A database was created containing 216 sets of data based on an experimental study. The database consists of parameters such as the percentage of cement substituted with granite powder, time of testing and curing conditions. It was shown that this method for designing green cementitious composite mixes, in terms of predicting compressive strength using ensemble models and only three input parameters, can be more accurate and much more precise than the conventional approach. Moreover, to the best of the authors' knowledge, artificial intelligence has been one of the most effective and precise methods used in the design and manufacturing industry in recent decades. The simplicity of this method makes it more suitable for construction practice due to the ease of evaluating the input variables. As the push towards decreasing carbon emissions increases, a method for designing green cementitious composites without producing waste that is more precise than traditional tests performed in a laboratory is essential.

## Introduction

The application of admixtures in the manufacturing of so-called 'green cementitious composites' has recently played a more important role in sustainable development. This is mainly due to the recent worldwide trend towards reducing the amount of carbon dioxide (CO_2_) generated during the production of Portland cement^1,2^. These composites are “green” because of the incorporation waste admixtures and as a partial replacement for cement. Such admixtures mainly include fly ash, ground granulated blast furnace slag (GGBFS), and granite powder^3–5^. An additional reason for their use is the fact that these materials are waste from various industrial processes^6^.

The use of granite powder as an admixture in mortars is mainly of interest because this material is difficult to recycle. Usually, this waste mineral is stored but has a decomposition time greater than 1,000,000 years. Granite is extremely hazardous in powder form because the powder particles are often suspended in the air and enter soil and water. Thus, mineral waste powders have the potential to cause respiratory failure in humans and animals. Furthermore, its disposal leads to water pollution and plant pollination (which is detrimental to the environment). The incorporation of waste mineral powders into solid material (such as mortar or concrete) reduces its hazardous effects, mitigating their harmfulness^7^. Recently, an increasing number of studies have focused on the behaviour of cementitious composites containing granite powder. This research is particularly related to the mechanical properties of hardened cementitious composites (e.g., compressive strength^8^, flexural strength^9^, tensile splitting strength^10^).

The conventional methodology for identifying the compressive strength of cementitious composites requires destructive laboratory tests. Unfortunately, these tests are very costly and time-consuming. For example, in the European Union, it costs no less than 100 euros to test one series of composites. Because these tests are destructive, they are performed on a limited number of samples, which may lead to imprecise results. This makes the conventional methodology ineffective and increases the carbon footprint of the process of obtaining mechanical properties. Furthermore, because with traditional methods^11^, the ability to evaluate the compressive strength of mortar containing a large amount (above 15% of the cement mass) of granite powder as a substitute for cement is lacking, a more precise method is needed. To the best of the authors' knowledge, artificial intelligence has been one of the most effective and precise methods used in the design and manufacturing industry in recent decades.

To overcome the disadvantages mentioned above, modelling methods based on machine learning algorithms are used more frequently to address various engineering problems (e.g., the prediction of the compressive strength^12^, adhesion between cementitious composite layers^13^, soil compression coefficient^14^, soil erosion susceptibility^15^, and axial compression capacity^16^ and the design of concrete mixtures^17^). Such modelling, using machine learning, consists of 5 steps: problem definition, data collection, modelling, evaluation and result analysis^18^.

Of these techniques, artificial neural networks (ANNs) are especially popular. In previous research, ANNs have been very useful for predicting the compressive strength of cementitious composites^19–21^ and have been used to determine the compressive strength of lightweight cementitious composites^22^ and cementitious bricks^23^, as well as the compressive strength of self-compacting composites^24^. However, such research is still needed for green cementitious composites containing different admixtures. First, the behaviour of such composites under various loading conditions should be determined. In particular, these admixtures affect the compressive strength of composites. Additionally, because creating machine learning-based models for compressive strength prediction is a nondestructive way of identifying these properties, it would reduce the associated costs and time for companies producing concrete. Daily, tons of concrete are wasted because of compressive strength tests due to the requirements of the standards imposed and the obligation to test all parts of a hardened concrete mixture, in some cases once a day^25^.

However, more recently, studies have been undertaken to use ensemble models due to their very high precision and good performance in predicting the compressive strength of concrete. This is because a random forest rejects linear assumptions and is able to learn the importance of inconsistent variables in datasets^26^. Notably, most ensemble models are characterized by higher overfitting resistance. Therefore, various ensemble models have been used, e.g., for the prediction of the compressive strength of cementitious composites^27^. These models have also been used successfully to predict the compressive strength of cementitious composites containing recycled rubber^28^, blast furnace slag, silica fume^29^ and fly ash^30^.

However, ensemble models for predicting the compressive strength of cementitious composites (cement paste, mortar, or concrete) where the cement is replaced by the waste granite powder are still lacking. This is a research gap that should be filled and is a goal of this article.

## Materials and methods

### Mixing proportions

**Figure 1:**
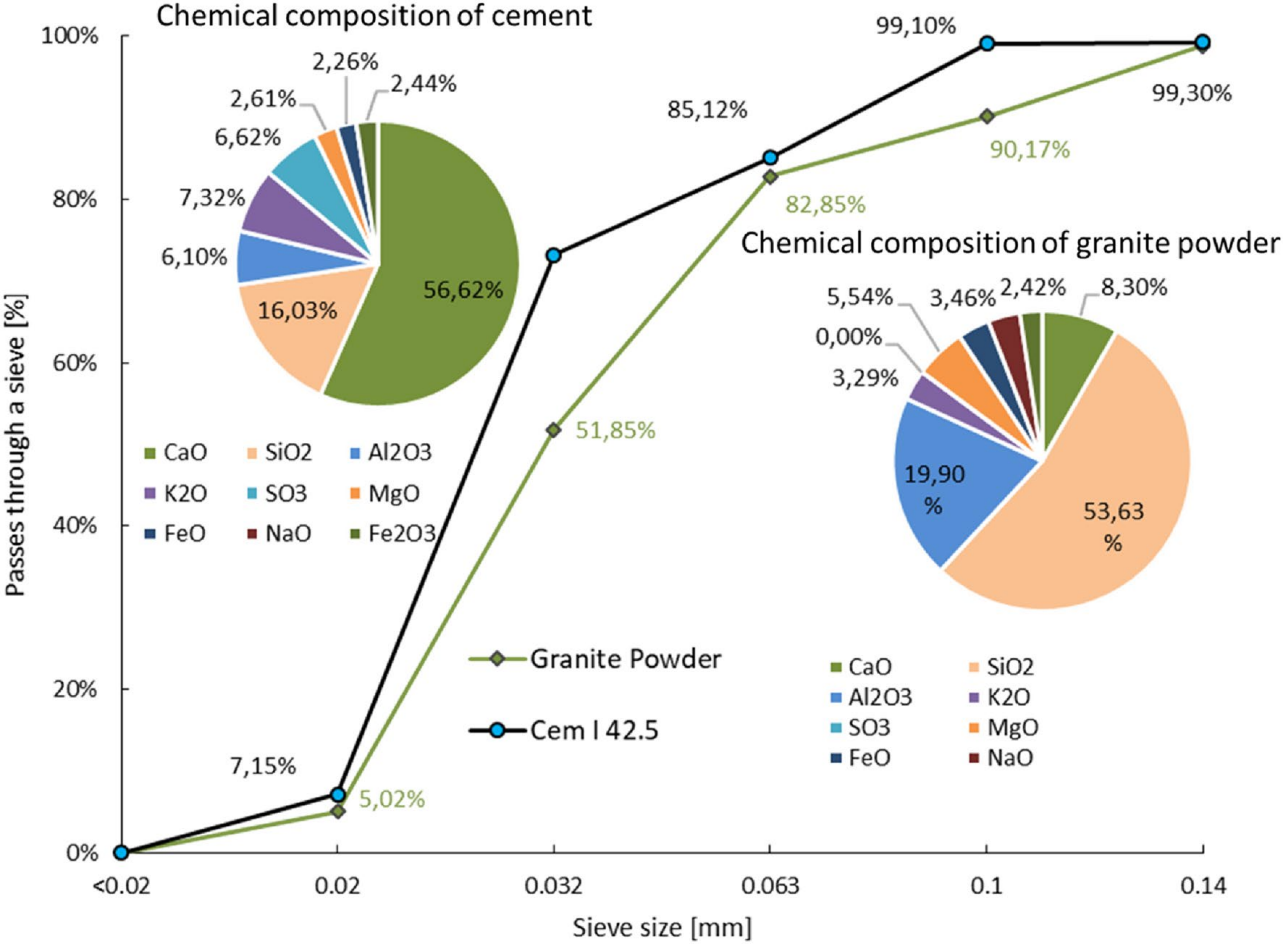
Comparison of the chemical properties and particle size distributions of ordinary Portland cement and granite powder.

In this study, ordinary Portland cement (OPC) and granite powder (GP) were used as binders. The physical and chemical properties of the cement and granite powder are described in Table 1 and Fig. 1, respectively. The particle size distributions of ordinary Portland cement and granite powder were investigated by means of the sieve size development method. Both materials were placed on a sieve assembly and then shaken for 180 s. Then, the residue on each sieve was weighed, and a screening curve was created. The particle size distributions of ordinary Portland cement and granite powder are compared in Fig. 1. River sand with a fineness modulus of 2.40, a specific gravity of 2.45, and a water absorption of 0.82 was used as the fine aggregate. In the present investigation, potable water was used for mixing and curing.

**Table 1:** Comparison of the physical properties of the cement and granite powder.

Characteristic	Units	Ordinary Portland Cement CEM I 42.5 R	Granite powder
Blaine’s fineness	m^2^/kg	365	395
Specific gravity	–	3.15	3.20
Initial setting time	minutes	105	220*
Final setting time	minutes	180	350*

*For cementitious composites with 30% of the cement replaced with granite powder.

In this research, 4 series of cement mortars were prepared, the compositions of which differed in the amount of cement replaced with granite powder (GP). Details of the proportions of the mortar mix by weight used in this study are presented in Table 2.

**Table 2:** Mixture proportions used in this study.

No	Cement (–)	Granite powder (–)	Fine aggregate (–)	Water/binder ratio	Water/cement ratio	Novikov slump (mm)
1	1.00	0	3.0	0.5	0.50	110
2	0.90	0.10			0.56	108
3	0.80	0.20			0.63	106
4	0.70	0.30			0.71	103

### Experimental program

**Figure 2:**
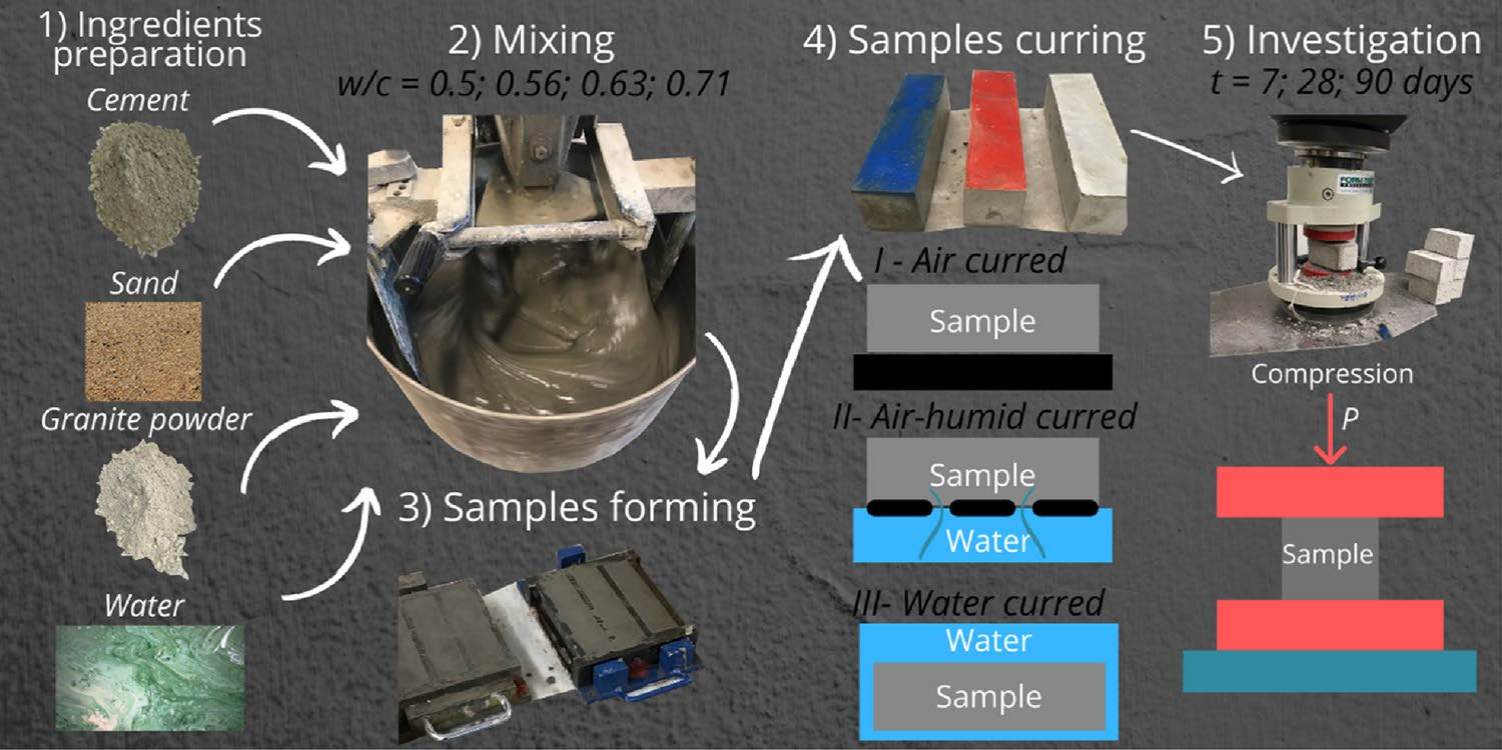
Production process of cement mortars with granite powder.

Figure 2 presents the research procedure. First, the dry ingredients were placed in a mixer and mixed for 30 s. Then, water was added, and the mixture was mixed for 90 s. Next, the mortar remnants on the walls of the mixer were manually peeled off, and the mixture was mixed for 90 s.

After mixing, the consistency of the mortar was investigated using the mortar slump subsidence method^31^, and then the mortar was placed in prepared forms. Twenty-four hours after moulding, the sample curing process started. The samples were divided into 3 groups and then stored according to the conditions described in Table 3.

**Table 3:** Curing conditions for cementitious mortar samples.

Description (–)	Type of curing (–)	Humidity (%)	Temperature (°C)
CC1	Air cured	20–55	16–30
CC2	Humid-air cured	55–90	23 ± 1
CC3	Water cured	100	23 ± 1

After 7, 28, and 90 days of curing, the samples were investigated by a compressive strength test. Compressive strength tests were performed using a compression strength test machine (Fig. 2) according to^32^.

## Results

### Statistical analyses of the obtained results

**Figure 3:**
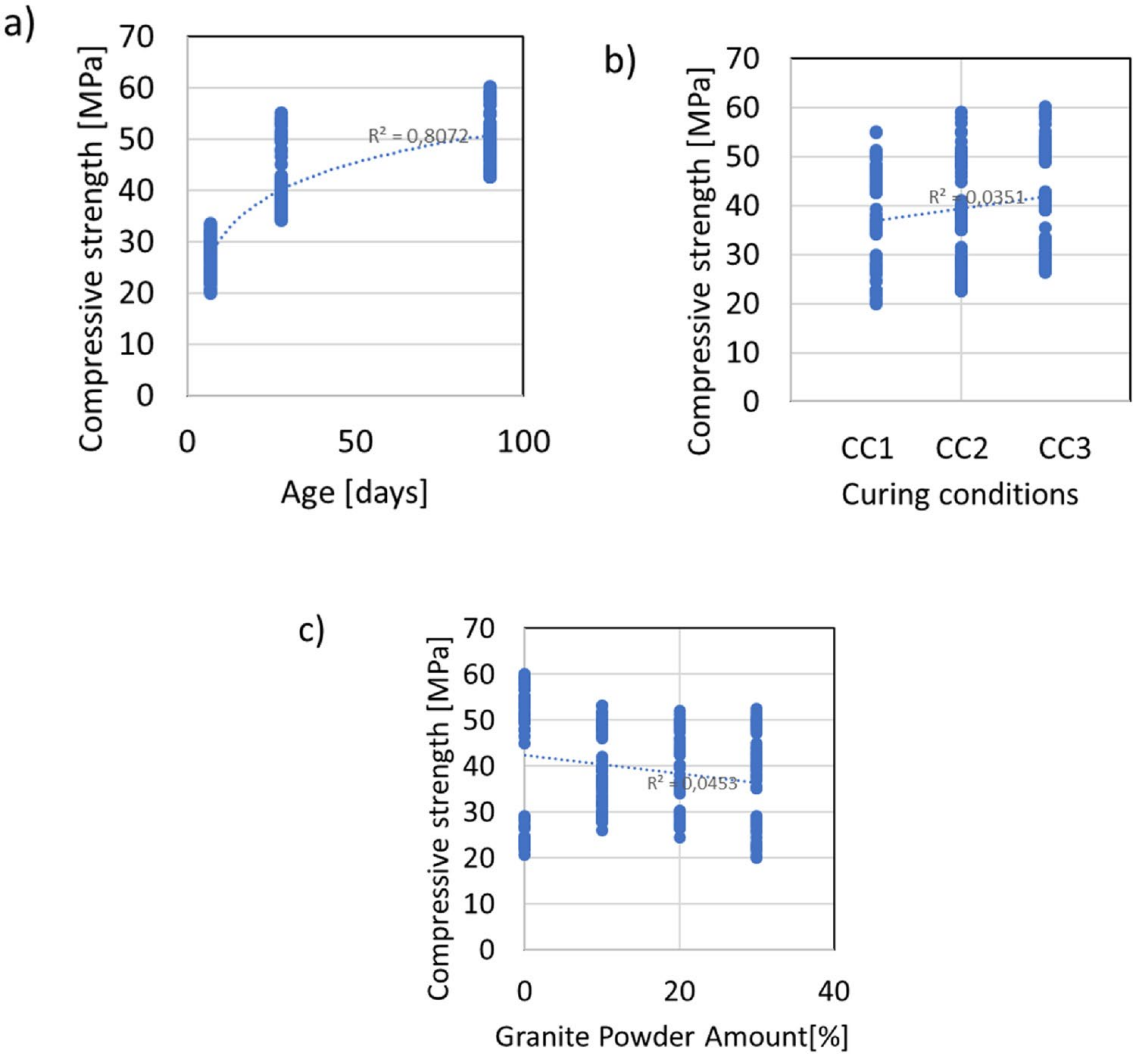
The relations between the compressive strength and (**a**) age, (**b**) curing conditions and (**c**) granite powder amount.

In the experimental program, only three variables were varied: age (7, 28 and 90 days), curing conditions (air cured, humid-air cured, and water cured) and water to cement ratio (0.5, 0.56, 0.63, and 0.71) as an expression of the decreasing amount of cement and increasing amount of granite powder. Thus, because the compression tests were performed on 2 halves after the tensile strength tests, the overall number of investigated samples was 216. In Fig. 3, the results of the compressive strength are presented with respect to age, curing conditions, and water-to-cement ratio.

According to Fig. 3, there is only a correlation between age and compressive strength. This is supported by the value of the coefficient of determination, which is equal to *R*^2^ = 0.807. For the other variables and the compressive strength, there is a lack of correlation, as evidenced by the very low values of the coefficient of determination, which are less than *R*^2^ = 0.4. As expected, the highest compressive strength values are obtained for the samples that were kept in water; their curing conditions are denoted as CC1. The older the samples are, the higher the value of the compressive strength obtained. However, the addition of the granite powder is unable to obtain compressive strength values equal to the 60 MPa of the reference sample, but due to the filling effect of the powder, the minimum values of compressive strength increase with increasing granite powder content (from approximately 20 MPa to 28 MPa for 10% replacement of cement by granite powder and to 25 MPa for 20% replacement of cement by granite powder). This effect is very promising for the design of low-quality cementitious composite mixtures.

### Modelling the compressive strength by means of ensemble models

As mentioned above, there are no strong correlation between the variables that are components of the mixture proportions, curing conditions, or testing age and compressive strength. Thus, it is reasonable to perform numerical analyses using more sophisticated techniques, e.g., ensemble models.

These models based on decision trees, which are considered supervised machine learning algorithms, are able to solve both regression and classification problems. The structure of such a decision tree consists of nodes in which a binary decision is made, and this division continues until the moment the algorithm is not able to separate the data in the node^33^. This node, called the leaf of the tree, provides the solution of the problem. The advantage of using this type of algorithm is the simplicity of the model obtained. However, in contrast, this is also a disadvantage because it might lead to algorithm overfitting. Decision trees are accurate and perform well on datasets with large variations in variables and when the number of records is not large^34^.

This problem might be solved by using a random forest algorithm, which uses many decision trees to obtain the solution to one problem. Each tree in the forest is built by a random training set, and at each node, division is carried out based on input variables that are randomly selected^35^.

**Figure 4:**
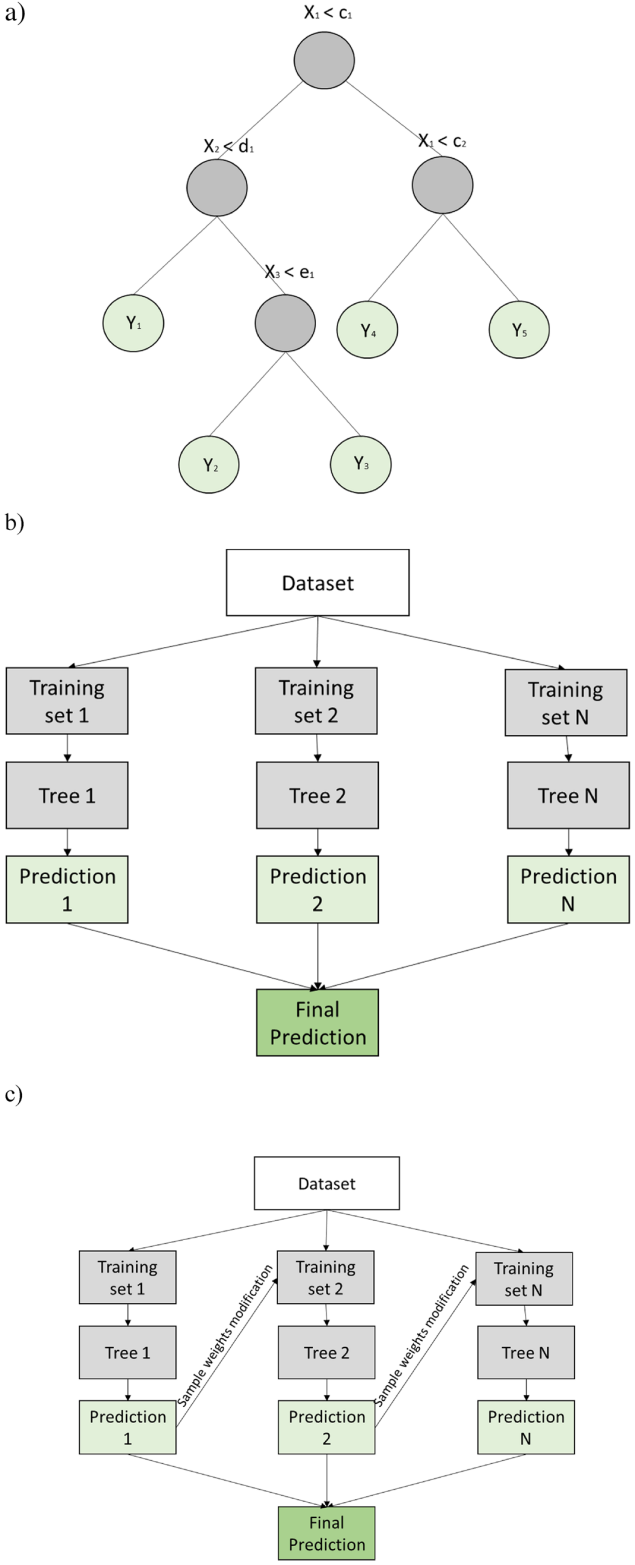
Schemes of ensemble models: (**a**) decision tree, (**b**) random forest and (**c**) AdaBoost.

However, in some cases, the performance of the random forest algorithm is not accurate, and efforts to improve it should be made. For this purpose, of the various ensemble learning algorithms, the adaptive boosting (AdaBoost) algorithm is the most typical and widely used^36^. This algorithm is effective because the next tree in the algorithm is modified based on the precision of the previous tree, strengthening the learning ability. The structural scheme of a decision tree, where the input variables are denoted *X*_i_ and the output variable is denoted *Y*_i_, is presented in Fig. 4 combined with the random forest and AdaBoost algorithm schemes.

The level of precision of the models is evaluated using a few parameters, which, according to^37^, can include the linear correlation coefficient (*R*), mean absolute error (*MAE*), root mean squared error (RMSE), and mean average percentage error (*MAPE*). The calculations of these parameters are performed as follows:

1 $$ R = \sqrt {1 - \frac{{\sum \left( {y - \hat{y}} \right)^{2} }}{{\sum \left( {y - \overline{y}} \right)^{2} }}} $$

2 $$ MAE = \frac{1}{n}\sum \left| {y - \hat{y}} \right| $$

3 $$ RMSE = \sqrt {\frac{{\sum \left( {y - \hat{y}} \right)^{2} }}{n}} $$

4 $$ MAPE = \frac{1}{n}\sum \left| {\frac{{y - \hat{y}}}{y}} \right| \cdot 100 $$

where *y*, measured value from the experimental test; $\hat{y}$, predicted value from the analyses; $\overline{y}$, mean value; *n*, number of data samples in the process.

**Figure 5:**
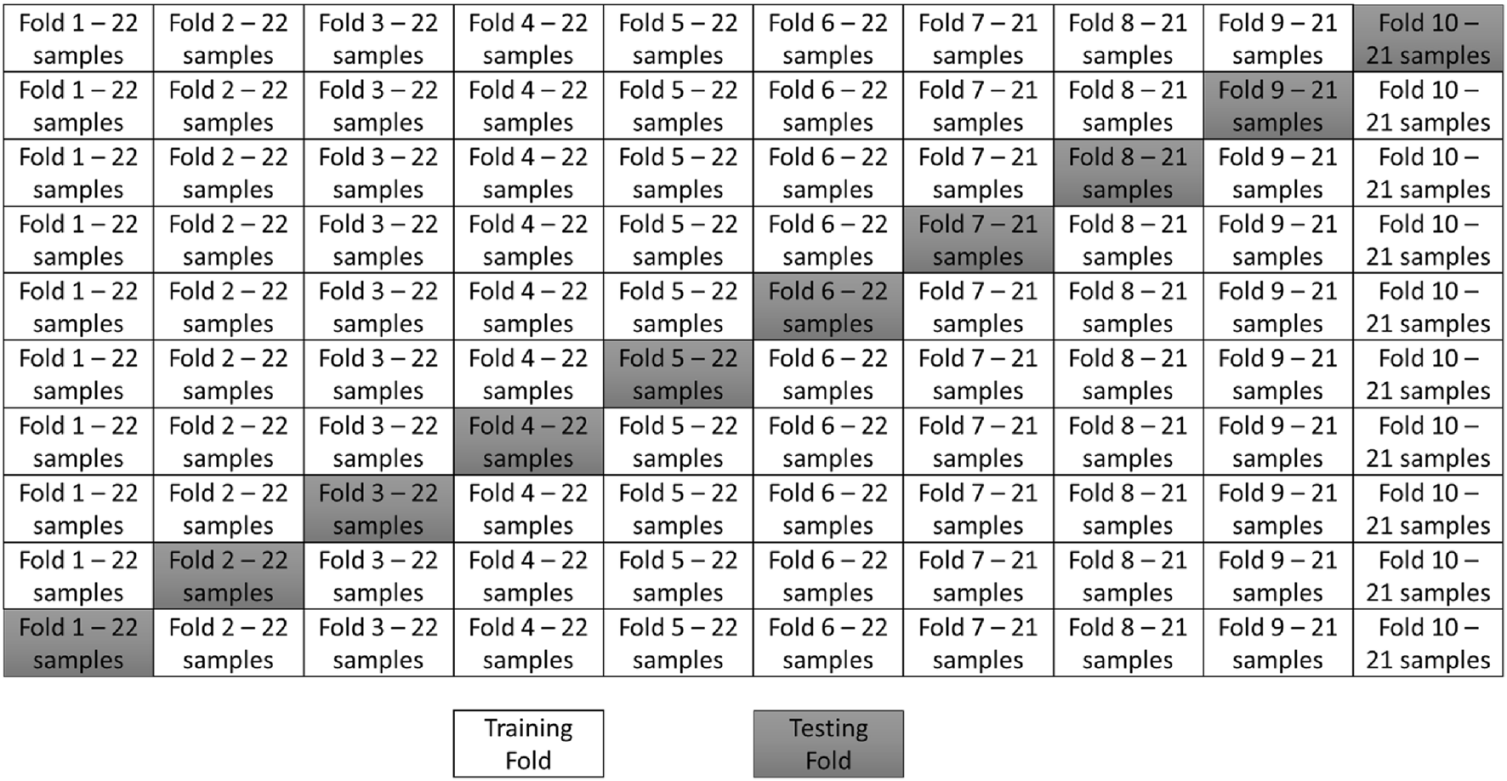
The division of the cross-validation folds.

Note that an *R* value closer to 1 corresponds to a better prediction from the algorithm. In turn, lower values of *MAE* and *RMSE* and *MAPE* mean that the algorithm predicts the output variables better than the other algorithms. Additionally, to avoid overfitting, tenfold cross-validation is performed according to^38^, as presented in Fig. 5.

**Figure 6:**
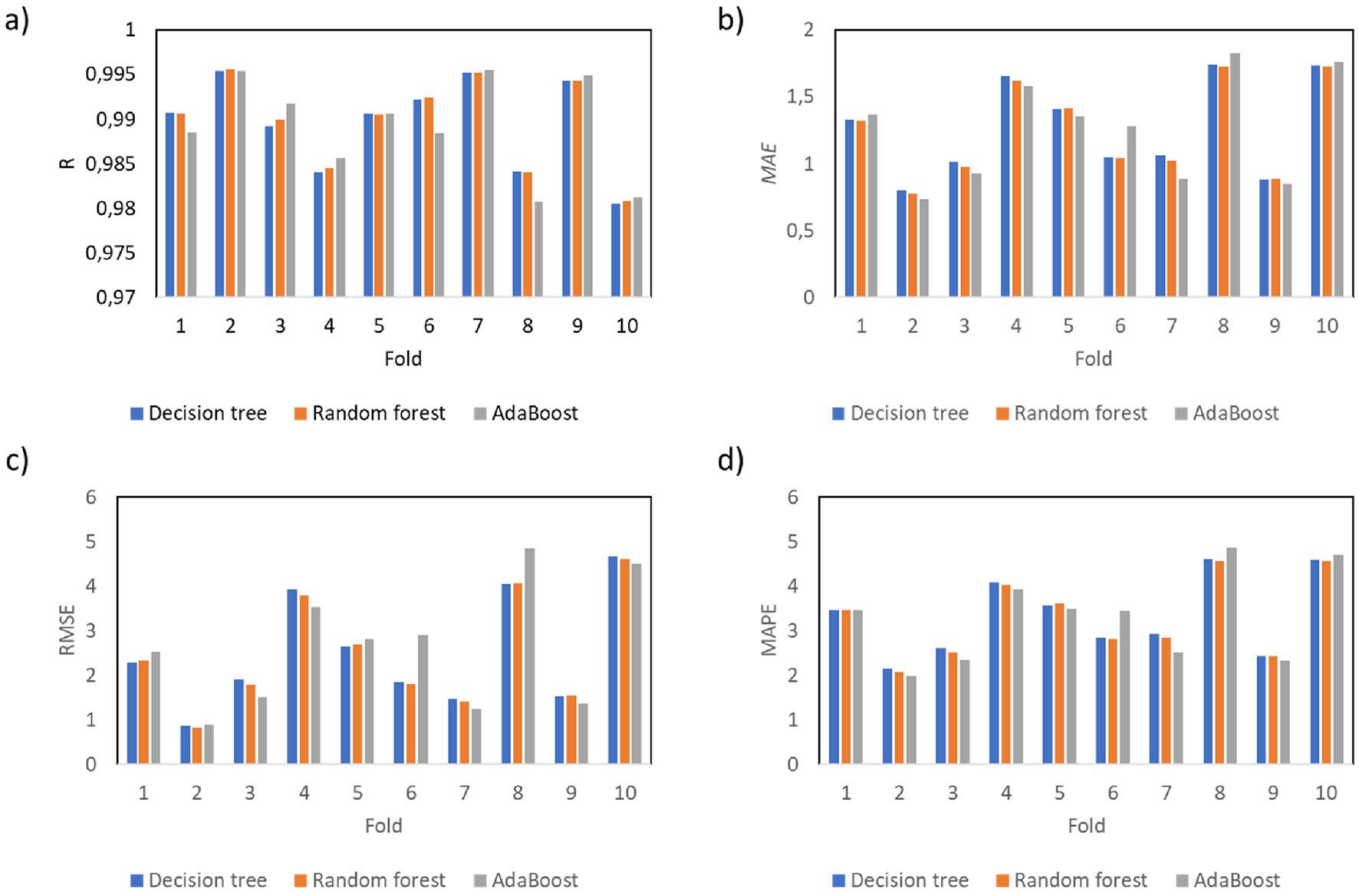
The performance of the analyses evaluated by (**a**) the linear coefficient of correlation, (**b**) mean average error, (**c**) root mean square error and (**d**) mean average percentage error.

**Figure 7:**
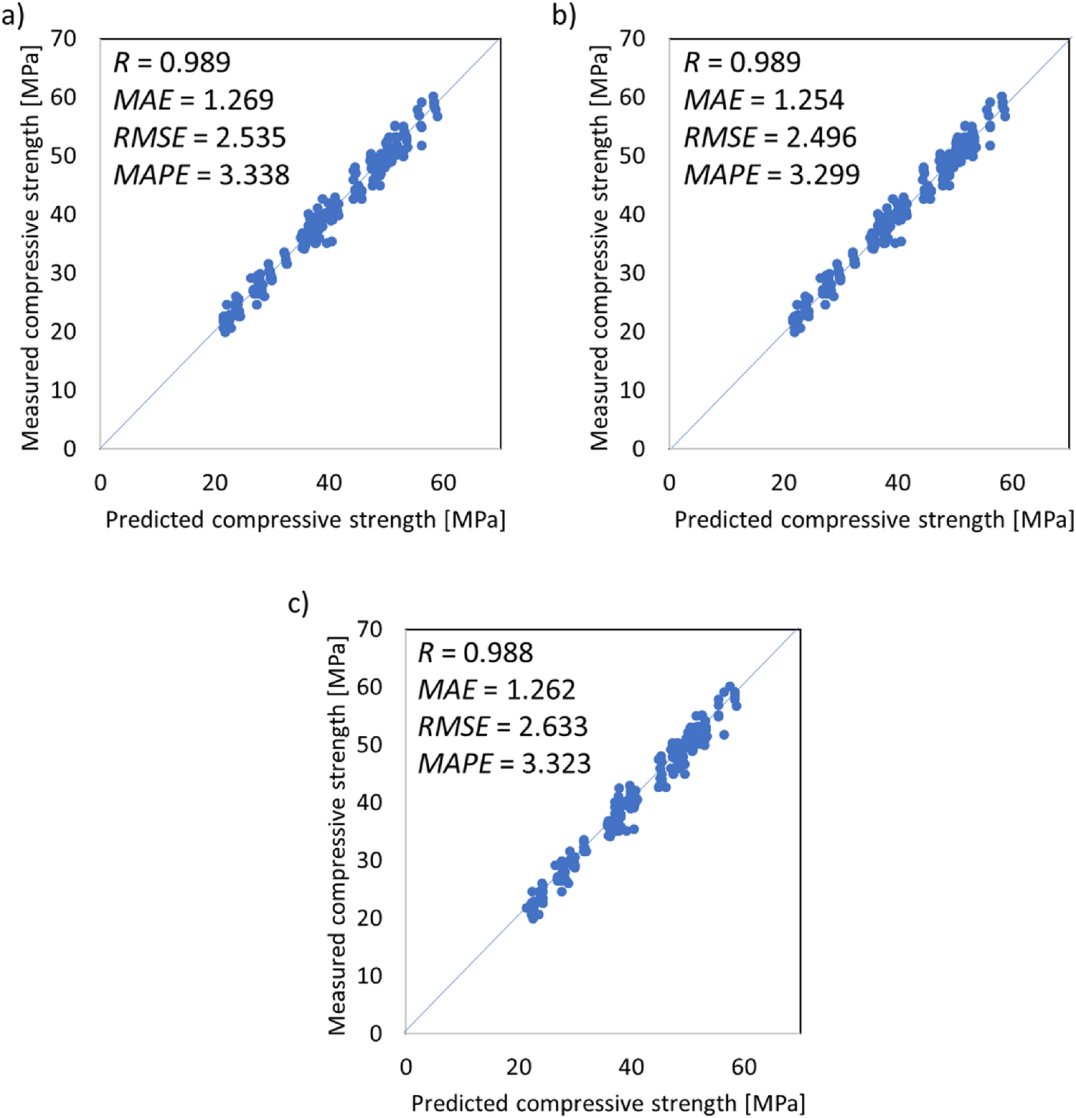
The relations between the measured compressive strength and predicted compressive strength by (**a**) decision tree, (**b**) random forest and (**c**) AdaBoost algorithms.

**Figure 8:**
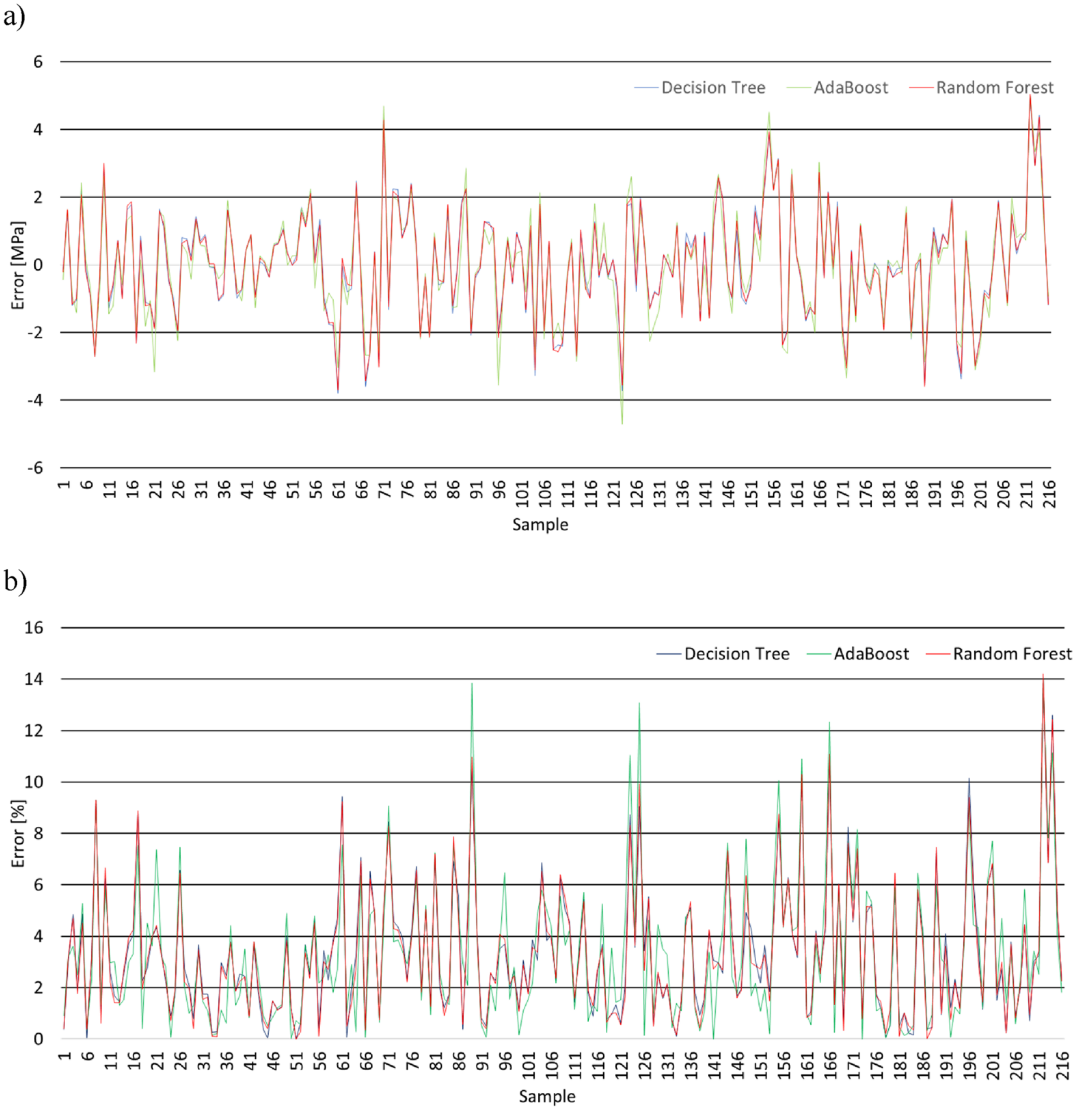
Prediction error distribution: (**a**) values and (**b**) percentage.

Based on the division of the dataset presented in Fig. 5, numerical analysis is performed. The performance of each fold is evaluated and presented in Fig. 6 in terms of the values of *R*, *MAE, RMSE* and *MAPE*. Moreover, the relations between the experimentally measured compressive strength value and those obtained using machine learning algorithms are presented in Fig. 7, combined with the error distribution in Fig. 8.

According to Figs. 6, 7 and 8, all of the investigated ensemble models are significantly precise in terms of predicting the compressive strength of mortar containing waste granite. This is evidenced by the very high values obtained for the linear correlation of coefficient *R*, which are close to 1.0. The accuracy of the performance is also supported by the very low errors values, which, as shown in Fig. 7, are less than 4%. Additionally, according to Fig. 8, the proposed models accurately predict the compressive strength values and only fail to properly predict the strength of a few samples (the percentage error is higher than 10%).

The proposed model is also accurate compared to other machine learning algorithms used for the purpose of predicting the compressive strength of green cementitious composites containing different admixtures. Some selected works are presented in Table 4 in addition to the results obtained by the models presented in this work.

**Table 4:** Comparison of algorithms used for compressive strength prediction of green cementitious composites containing different admixtures.

References	Type of admixture	Level of precision		Machine learning model
		Linear coefficient of correlation *R*	Root mean squared error *RMSE* [MPa]	
Han et al.^3^	Ground granulated blast furnace slag	0.984	2.47	PSO-BP
Ahmad et al.^12^	Fly ash	0.954	4.03	DT-Bagging
Behnood et al.^20^	Silica fume	0.988	3.93	HANNMOGW
Kandiri et al.^21^	Ground granulated blast furnace slag	0.980	2.12	MOANN
This work	Granite powder	0.989	2.54	DT
		0.989	2.50	RF
		0.988	2.63	AdaBoost

Analysis of the results in Table 4 shows that the levels of precision for the compressive strength of green cementitious composites using machine learning algorithms are very high. Additionally, in this work, a very precise model for predicting the compressive strength of green cementitious composite containing different admixtures, in comparison to those investigated previously, is constructed.

## Conclusions

In this article, a comparison of three ensemble models for predicting the compressive strength of mortars containing waste granite powder, taking into account the age of the samples and the curing conditions, was presented. For this purpose, a database was built based on an experimental program. This database was formulated on the basis of tests performed on standardized samples prepared and tested at different ages and cured under different conditions. Based on the presented research, the following conclusions can be drawn:

- The article shows that it is possible to predict the compressive strength of mortars with granite powder additions based on just three parameters: the testing age, the water to cement ratio and the curing conditions. Therefore, the presented method can be seen to be simple and reliable in use.
- The usefulness of this method was proven by the very high values of the linear correlation coefficient *R*, which equal 0.989 for the decision tree, 0.989 for the random forest and 0.988 for AdaBoost.
- All models were characterized by low error values, which in the case of *MAE* were less than 1.270 MPa, in the case of *RMSE* were less than 2.633 MPa, and in the case of *MAPE* were less than 3.35%.

The authors emphasize that the proposed method has limitations, which include the time of the test and the water-to-cement ratio. However, the only curing conditions that were not taken into account in this paper were characterized by high temperature; thus, these models can be used in almost any conditions under which samples are cured. From a practical point of view, it might be beneficial to verify whether this model is accurate for samples prepared by other researchers. Moreover, whether the model can be used for similar cementitious composite mixtures but with other waste mineral powders should be verified. Moreover, it might be beneficial to model other properties of green cementitious composites such as subsurface tensile strength, creep strain, or shrinkage. Moreover, due to the ecologically inspired push towards using waste materials in cementitious composites, constantly updating the model to make it suitable for newly designed cementitious composite mixtures would be reasonable.

## Supplementary Information

Supplementary Information.

## Data Availability

All data generated or analysed during this study are included in this published article and its supplementary information files.
