# Supplementary material for: Design of a machine learning model for the precise manufacturing of green cementitious composites modified with waste granite powder
Source: Sci Rep. 2022 Aug 2;12:13242. doi: 10.1038/s41598-022-17670-6 (PMC9345902; doi:10.1038/s41598-022-17670-6)
Supplement: Supplementary file 1 — Supplementary Information. [file 41598_2022_17670_MOESM1_ESM.docx]

Appendix 1

| Sample | w/c | Curing conditions | Testing Time | *f*_c_ | Sample | w/c | Curing conditions | Testing Time | *f*_c_ |
| --- | --- | --- | --- | --- | --- | --- | --- | --- | --- |
| 1 | 0.5 | CC1 | 7 | 22.89 | 44 | 0.5 | CC3 | 28 | 52.64 |
| 2 | 0.5 | CC1 | 7 | 24.58 | 45 | 0.5 | CC3 | 28 | 53.75 |
| 3 | 0.5 | CC1 | 7 | 20.68 | 46 | 0.5 | CC3 | 28 | 54.1 |
| 4 | 0.5 | CC1 | 7 | 22.48 | 47 | 0.5 | CC3 | 28 | 55.04 |
| 5 | 0.5 | CC1 | 7 | 21.86 | 48 | 0.5 | CC3 | 28 | 53.1 |
| 6 | 0.5 | CC1 | 7 | 22.51 | 49 | 0.5 | CC3 | 90 | 59.14 |
| 7 | 0.5 | CC1 | 28 | 49.5 | 50 | 0.5 | CC3 | 90 | 60.14 |
| 8 | 0.5 | CC1 | 28 | 46.6 | 51 | 0.5 | CC3 | 90 | 58.4 |
| 9 | 0.5 | CC1 | 28 | 44.98 | 52 | 0.5 | CC3 | 90 | 56.7 |
| 10 | 0.5 | CC1 | 28 | 47.98 | 53 | 0.5 | CC3 | 90 | 59.12 |
| 11 | 0.5 | CC1 | 28 | 50.1 | 54 | 0.5 | CC3 | 90 | 57.8 |
| 12 | 0.5 | CC1 | 28 | 47.8 | 55 | 0.56 | CC1 | 7 | 28.1 |
| 13 | 0.5 | CC1 | 90 | 55.14 | 56 | 0.56 | CC1 | 7 | 29.88 |
| 14 | 0.5 | CC1 | 90 | 50.8 | 57 | 0.56 | CC1 | 7 | 27.96 |
| 15 | 0.5 | CC1 | 90 | 54.96 | 58 | 0.56 | CC1 | 7 | 29.7 |
| 16 | 0.5 | CC1 | 90 | 50.87 | 59 | 0.56 | CC1 | 7 | 27.81 |
| 17 | 0.5 | CC1 | 90 | 51.2 | 60 | 0.56 | CC1 | 7 | 26.01 |
| 18 | 0.5 | CC1 | 90 | 49.9 | 61 | 0.56 | CC1 | 28 | 35.8 |
| 19 | 0.5 | CC2 | 7 | 24.8 | 62 | 0.56 | CC1 | 28 | 36.4 |
| 20 | 0.5 | CC2 | 7 | 24.1 | 63 | 0.56 | CC1 | 28 | 36.8 |
| 21 | 0.5 | CC2 | 7 | 23.1 | 64 | 0.56 | CC1 | 28 | 36.11 |
| 22 | 0.5 | CC2 | 7 | 22.8 | 65 | 0.56 | CC1 | 28 | 34.58 |
| 23 | 0.5 | CC2 | 7 | 23.9 | 66 | 0.56 | CC1 | 28 | 35.12 |
| 24 | 0.5 | CC2 | 7 | 24.1 | 67 | 0.56 | CC1 | 90 | 45.98 |
| 25 | 0.5 | CC2 | 28 | 50.48 | 68 | 0.56 | CC1 | 90 | 48.24 |
| 26 | 0.5 | CC2 | 28 | 51.25 | 69 | 0.56 | CC1 | 90 | 47.89 |
| 27 | 0.5 | CC2 | 28 | 53.1 | 70 | 0.56 | CC1 | 90 | 46.47 |
| 28 | 0.5 | CC2 | 28 | 49.8 | 71 | 0.56 | CC1 | 90 | 48.15 |
| 29 | 0.5 | CC2 | 28 | 50.1 | 72 | 0.56 | CC1 | 90 | 50.35 |
| 30 | 0.5 | CC2 | 28 | 50.42 | 73 | 0.56 | CC2 | 7 | 29.56 |
| 31 | 0.5 | CC2 | 90 | 56.8 | 74 | 0.56 | CC2 | 7 | 30.58 |
| 32 | 0.5 | CC2 | 90 | 59.1 | 75 | 0.56 | CC2 | 7 | 31.55 |
| 33 | 0.5 | CC2 | 90 | 55.1 | 76 | 0.56 | CC2 | 7 | 29.1 |
| 34 | 0.5 | CC2 | 90 | 54.89 | 77 | 0.56 | CC2 | 7 | 28.7 |
| 35 | 0.5 | CC2 | 90 | 57.89 | 78 | 0.56 | CC2 | 7 | 29.12 |
| 36 | 0.5 | CC2 | 90 | 51.8 | 79 | 0.56 | CC2 | 28 | 35.9 |
| 37 | 0.5 | CC3 | 7 | 26.9 | 80 | 0.56 | CC2 | 28 | 37.15 |
| 38 | 0.5 | CC3 | 7 | 28.1 | 81 | 0.56 | CC2 | 28 | 37.85 |
| 39 | 0.5 | CC3 | 7 | 26.79 | 82 | 0.56 | CC2 | 28 | 39.15 |
| 40 | 0.5 | CC3 | 7 | 26.42 | 83 | 0.56 | CC2 | 28 | 37.58 |
| 41 | 0.5 | CC3 | 7 | 29.14 | 84 | 0.56 | CC2 | 28 | 39.1 |
| 42 | 0.5 | CC3 | 7 | 28.79 | 85 | 0.56 | CC2 | 90 | 47.86 |
| 43 | 0.5 | CC3 | 28 | 51.48 | 86 | 0.56 | CC2 | 90 | 48.97 |
| Sample | w/c | Curing conditions | Testing Time | *f*_c_ | Sample | w/c | Curing conditions | Testing Time | *f*_c_ |
| 87 | 0.56 | CC2 | 90 | 46.87 | 132 | 0.63 | CC2 | 7 | 28.15 |
| 88 | 0.56 | CC2 | 90 | 49.12 | 133 | 0.63 | CC2 | 28 | 36.7 |
| 89 | 0.56 | CC2 | 90 | 48.59 | 134 | 0.63 | CC2 | 28 | 35.14 |
| 90 | 0.56 | CC2 | 90 | 49.58 | 135 | 0.63 | CC2 | 28 | 35.5 |
| 91 | 0.56 | CC3 | 7 | 31.58 | 136 | 0.63 | CC2 | 28 | 38.1 |
| 92 | 0.56 | CC3 | 7 | 33.57 | 137 | 0.63 | CC2 | 28 | 38.15 |
| 93 | 0.56 | CC3 | 7 | 33.14 | 138 | 0.63 | CC2 | 28 | 40.15 |
| 94 | 0.56 | CC3 | 7 | 32.18 | 139 | 0.63 | CC2 | 90 | 48.15 |
| 95 | 0.56 | CC3 | 7 | 31.58 | 140 | 0.63 | CC2 | 90 | 44.86 |
| 96 | 0.56 | CC3 | 7 | 32.28 | 141 | 0.63 | CC2 | 90 | 45.89 |
| 97 | 0.56 | CC3 | 28 | 39.14 | 142 | 0.63 | CC2 | 90 | 49.15 |
| 98 | 0.56 | CC3 | 28 | 38.94 | 143 | 0.63 | CC2 | 90 | 48.95 |
| 99 | 0.56 | CC3 | 28 | 39.88 | 144 | 0.63 | CC2 | 90 | 47.98 |
| 100 | 0.56 | CC3 | 28 | 40.14 | 145 | 0.63 | CC3 | 7 | 29.18 |
| 101 | 0.56 | CC3 | 28 | 41.23 | 146 | 0.63 | CC3 | 7 | 30.15 |
| 102 | 0.56 | CC3 | 28 | 41.98 | 147 | 0.63 | CC3 | 7 | 30.35 |
| 103 | 0.56 | CC3 | 90 | 50.14 | 148 | 0.63 | CC3 | 7 | 30.17 |
| 104 | 0.56 | CC3 | 90 | 51.58 | 149 | 0.63 | CC3 | 7 | 29.58 |
| 105 | 0.56 | CC3 | 90 | 51.08 | 150 | 0.63 | CC3 | 7 | 28.95 |
| 106 | 0.56 | CC3 | 90 | 53.14 | 151 | 0.63 | CC3 | 28 | 39.7 |
| 107 | 0.56 | CC3 | 90 | 53.11 | 152 | 0.63 | CC3 | 28 | 35.47 |
| 108 | 0.56 | CC3 | 90 | 51.89 | 153 | 0.63 | CC3 | 28 | 39.51 |
| 109 | 0.63 | CC1 | 7 | 26.89 | 154 | 0.63 | CC3 | 28 | 40.28 |
| 110 | 0.63 | CC1 | 7 | 27.15 | 155 | 0.63 | CC3 | 28 | 40.18 |
| 111 | 0.63 | CC1 | 7 | 24.59 | 156 | 0.63 | CC3 | 28 | 42.58 |
| 112 | 0.63 | CC1 | 7 | 29.1 | 157 | 0.63 | CC3 | 90 | 48.9 |
| 113 | 0.63 | CC1 | 7 | 26.9 | 158 | 0.63 | CC3 | 90 | 52.15 |
| 114 | 0.63 | CC1 | 7 | 26.45 | 159 | 0.63 | CC3 | 90 | 51.18 |
| 115 | 0.63 | CC1 | 28 | 35.14 | 160 | 0.63 | CC3 | 90 | 49.75 |
| 116 | 0.63 | CC1 | 28 | 36.78 | 161 | 0.63 | CC3 | 90 | 50.16 |
| 117 | 0.63 | CC1 | 28 | 34.15 | 162 | 0.63 | CC3 | 90 | 49.99 |
| 118 | 0.63 | CC1 | 28 | 36.17 | 163 | 0.71 | CC1 | 7 | 20.58 |
| 119 | 0.63 | CC1 | 28 | 35.98 | 164 | 0.71 | CC1 | 7 | 22.18 |
| 120 | 0.63 | CC1 | 28 | 34.31 | 165 | 0.71 | CC1 | 7 | 19.95 |
| 121 | 0.63 | CC1 | 90 | 45.98 | 166 | 0.71 | CC1 | 7 | 22.56 |
| 122 | 0.63 | CC1 | 90 | 44.25 | 167 | 0.71 | CC1 | 7 | 21.7 |
| 123 | 0.63 | CC1 | 90 | 47.5 | 168 | 0.71 | CC1 | 7 | 22.53 |
| 124 | 0.63 | CC1 | 90 | 43.58 | 169 | 0.71 | CC1 | 28 | 38.12 |
| 125 | 0.63 | CC1 | 90 | 43.15 | 170 | 0.71 | CC1 | 28 | 37.01 |
| 126 | 0.63 | CC1 | 90 | 42.61 | 171 | 0.71 | CC1 | 28 | 35.08 |
| 127 | 0.63 | CC2 | 7 | 28.15 | 172 | 0.71 | CC1 | 28 | 39.22 |
| 128 | 0.63 | CC2 | 7 | 29.58 | 173 | 0.71 | CC1 | 28 | 37.95 |
| 129 | 0.63 | CC2 | 7 | 27.41 | 174 | 0.71 | CC1 | 28 | 35.51 |
| 130 | 0.63 | CC2 | 7 | 26.58 | 175 | 0.71 | CC1 | 90 | 48.1 |
| 131 | 0.63 | CC2 | 7 | 27.15 | 176 | 0.71 | CC1 | 90 | 47.12 |
| Sample | w/c | Curing conditions | Testing Time | *f*_c_ | Sample | w/c | Curing conditions | Testing Time | *f*_c_ |
| 177 | 0.71 | CC1 | 90 | 44.08 |  |  |  |  |  |
| 178 | 0.71 | CC1 | 90 | 43.95 |  |  |  |  |  |
| 179 | 0.71 | CC1 | 90 | 44.95 |  |  |  |  |  |
| 180 | 0.71 | CC1 | 90 | 42.71 |  |  |  |  |  |
| 181 | 0.71 | CC2 | 7 | 22.59 |  |  |  |  |  |
| 182 | 0.71 | CC2 | 7 | 24.58 |  |  |  |  |  |
| 183 | 0.71 | CC2 | 7 | 26.04 |  |  |  |  |  |
| 184 | 0.71 | CC2 | 7 | 25.58 |  |  |  |  |  |
| 185 | 0.71 | CC2 | 7 | 23.42 |  |  |  |  |  |
| 186 | 0.71 | CC2 | 7 | 22.58 |  |  |  |  |  |
| 187 | 0.71 | CC2 | 28 | 37.95 |  |  |  |  |  |
| 188 | 0.71 | CC2 | 28 | 39.15 |  |  |  |  |  |
| 189 | 0.71 | CC2 | 28 | 35.15 |  |  |  |  |  |
| 190 | 0.71 | CC2 | 28 | 39.85 |  |  |  |  |  |
| 191 | 0.71 | CC2 | 28 | 39.9 |  |  |  |  |  |
| 192 | 0.71 | CC2 | 28 | 41.05 |  |  |  |  |  |
| 193 | 0.71 | CC2 | 90 | 50.15 |  |  |  |  |  |
| 194 | 0.71 | CC2 | 90 | 50.35 |  |  |  |  |  |
| 195 | 0.71 | CC2 | 90 | 49.85 |  |  |  |  |  |
| 196 | 0.71 | CC2 | 90 | 48.1 |  |  |  |  |  |
| 197 | 0.71 | CC2 | 90 | 49.05 |  |  |  |  |  |
| 198 | 0.71 | CC2 | 90 | 48.95 |  |  |  |  |  |
| 199 | 0.71 | CC3 | 7 | 27.18 |  |  |  |  |  |
| 200 | 0.71 | CC3 | 7 | 27.96 |  |  |  |  |  |
| 201 | 0.71 | CC3 | 7 | 29.25 |  |  |  |  |  |
| 202 | 0.71 | CC3 | 7 | 29.01 |  |  |  |  |  |
| 203 | 0.71 | CC3 | 7 | 26.95 |  |  |  |  |  |
| 204 | 0.71 | CC3 | 7 | 27.95 |  |  |  |  |  |
| 205 | 0.71 | CC3 | 28 | 42.15 |  |  |  |  |  |
| 206 | 0.71 | CC3 | 28 | 42.9 |  |  |  |  |  |
| 207 | 0.71 | CC3 | 28 | 41.8 |  |  |  |  |  |
| 208 | 0.71 | CC3 | 28 | 40.95 |  |  |  |  |  |
| 209 | 0.71 | CC3 | 28 | 40.57 |  |  |  |  |  |
| 210 | 0.71 | CC3 | 28 | 39.74 |  |  |  |  |  |
| 211 | 0.71 | CC3 | 90 | 52.58 |  |  |  |  |  |
| 212 | 0.71 | CC3 | 90 | 50.7 |  |  |  |  |  |
| 213 | 0.71 | CC3 | 90 | 50.4 |  |  |  |  |  |
| 214 | 0.71 | CC3 | 90 | 49.2 |  |  |  |  |  |
| 215 | 0.71 | CC3 | 90 | 49.05 |  |  |  |  |  |
| 216 | 0.71 | CC3 | 90 | 51.56 |  |  |  |  |  |
